# Editorial: Immune plasticity in mixed pattern rheumatic diseases

**DOI:** 10.3389/fimmu.2025.1665204

**Published:** 2025-08-22

**Authors:** Matilde Otero-Losada, Rodolfo Kölliker-Frers, Radjesh Bisoendial, Francisco Capani

**Affiliations:** ^1^ Centro de Altos Estudios en Ciencias Humanas y de la Salud, Universidad Abierta Interamericana, Consejo Nacional de Investigaciones Científicas y Técnicas, CAECIHS, UAI-CONICET, Buenos Aires, Argentina; ^2^ Department of Rheumatology and Clinical Immunology, Maasstad Hospital, Rotterdam, Netherlands; ^3^ Department of Developmental Biology, Erasmus Medical Center, Rotterdam, Netherlands; ^4^ Instituto de Ciencias Biomédicas, Facultad de Ciencias de la Salud, Universidad Autónoma de Chile, Santiago, Chile

**Keywords:** T lymphocyte phenotypes, Th-17, autoreactive effector cells, autoantibodies, effector cytokines, regulatory cytokines and effector molecules, immune tolerance

Scientific evidence supports the crucial role of immune system plasticity, the ability of the immune system to undergo phenotypic and functional changes in the borderline zone between restoration and homeostasis failure. Immune plasticity involves phenotypic variability following both internal and external environmental challenges. Autoinflammatory and autoimmune diseases are spread over an immunological continuum, ranging from autoinflammatory disorders with inflammation and no autoantibodies or autoreactive T lymphocytes to autoimmune disorders with autoantibodies and autoreactive T lymphocytes. The interaction between innate and adaptive immunity likely influences immunopathogenic plasticity, affecting both ends of the immunological spectrum.

Autoimmunity and autoinflammation are both expressions of an aberrant immune response to the host components and give rise to various diseases, characterized by immune dysfunction. Immune tolerance towards host constituent elements is essential in maintaining immune homeostasis and preventing undesired autoimmune pathology. Thus, unimpaired cellular and humoral immune responses avoid self-recognition and obliterate pathogens. Yet, perpetuated immune hypersensitivity reactions and other less-defined injury mechanisms may lead to disease. Immune-induced damage may be amplified due to a dysregulated inflammatory response with a remitting-relapsing or progressive pattern. The goal of this topic is to discuss how immune plasticity participates in the pathophysiology, evolution, and treatment of mixed autoinflammatory and autoimmune rheumatic disease. A few interesting studies are presented.

Treg Cell Plasticity as a Driver of Inflammation in Spondyloarthritis and Psoriasis. The authors discuss lineage plasticity of regulatory T cells (Tregs), that are known for their ability to maintain immune tolerance by suppressing effector T cell responses. Yet, in chronic inflammatory diseases as spondyloarthritis (SpA) and psoriasis (PsO), Tregs acquire proinflammatory characteristics. Under inflammatory conditions, Tregs downregulate FoxP3, upregulate RORγt, and produce IL-17A and IFN-γ cytokines, losing their suppressive function and aggravating disease. In SpA, impaired Treg function has been identified in peripheral blood and synovial fluid. Specific subsets like CD161 Tregs with Th17-like features suggest that inflammatory cytokines and signals like STAT3 activation and ICOS engagement promote pathogenic reprogramming. Genetic factors like HLA-B27 may further predispose Tregs to instability. Single-cell transcriptome analyses have shown shared TCR repertoires between Tregs and effector T cells, reinforcing the idea of lineage plasticity. In PsO, skin-resident Tregs exposed to IL-23, IL-6, and IL-21 can acquire a Th17-like phenotype, producing IL-17A and exacerbating local inflammation. Environmental factors as hypoxia also contribute to destabilizing Treg identity. The persistence of pathogenic Tregs, even following therapeutic blockade of IL-17A or IL-23, highlights the challenge of achieving long-term disease remission (1, 2).

Molecular genetics in adult-onset Still’s disease: next-generation sequencing in 24 patients and literature review. Adult-onset Still’s disease (AOSD) is a rare systemic inflammatory disease characterized by intermittent fever, arthritis, and evanescent skin rash in the absence of infection, malignancy, or rheumatological disease. Growing evidence supports that Adult-onset Still’s disease (AOSD) is an autoinflammatory disorder where the innate immune system plays a pivotal role. Although AOSD shares common clinical and pathogenic features with other systemic autoinflammatory diseases (SAIDs), its genetic profile seems to be different. Next-generation sequencing (NGS) panels are increasingly used for the diagnosis of monogenic systemic autoinflammatory diseases (SAIDs). However, their role in patients with adult-onset Still’s disease (AOSD) remains unknown. The authors performed an observational, multicenter study, examining 24 patients (16 men, 8 women, average 42.2 ± 17.9 y.o.) with AOSD diagnosis who underwent NGS panel testing to evaluate the usefulness of NGS panels to improve AOSD diagnosis and management. Clinical manifestations, laboratory parameters, complications, and therapeutic responses were recorded. Overall, NGS was useful in ruling out the presence of pathogenic genetic variants related to other SAIDs. Their most interesting results are discussed here (3, 4).

Gut microbiome and metabolomics in systemic sclerosis: feature, link and mechanisms. Systemic sclerosis (SSc) is a rare and highly heterogeneous chronic autoimmune disease characterized by multiple organ malfunction caused by fibrosis, often accompanied by a poor prognosis and high mortality rates. The primary pathogenic mechanisms of SSc involve tissue fibrosis, autoimmune dysfunction, and microvascular abnormalities. Recent studies have shed light on the gut microbiota (GM) and metabolites in SSc patients, revealing their association with gastrointestinal symptoms and disease phenotypes. Yet, the specific mechanisms underlying the interactions between GM, metabolites, and the immune system and their roles in the pathogenesis of SSc are still unclear. The authors outline the characteristics of GM and metabolites in SSc patients, exploring their interrelationship and analyzing their correlation with clinical SSc phenotypes. They discuss interesting differences in bacterial diversity and the abundance of specific bacterial genera, closely linked to gastrointestinal symptoms, and also in the levels of amino acids and lipid metabolites compared with healthy individuals. Integrated multi-omics data analysis provides a more comprehensive understanding of DNA identification and metabolite functions in the microbiome, thus enhancing the informative value of microbial research in SSc (5, 6).

Neutrophil exhaustion and impaired functionality in psoriatic arthritis patients. Neutrophils (polymorphonuclear leukocytes, PMNs) are the most abundant subtype of white blood cells and are among the main actors of the innate inflammatory response. Psoriatic arthritis (PsA) is a chronic inflammatory disease affecting both the axial and peripheral joints. Typically associated with psoriasis, PsA can also affect multiple systems and organs, including the nails, entheses, eyes and cardiovascular system. Despite previous observations concerning the involvement of PMNs in PsA, their specific role in the disease remains poorly understood. This study aimed to characterize the biological functions of PMNs and neutrophil-related mediators in PsA patients. The authors studied 31 PsA patients and 22 healthy controls (HCs). PMNs isolated from peripheral blood were subjected to *in vitro* stimulation with lipopolysaccharide (LPS), N-Formylmethionyl-leucyl-phenylalanine (fMLP), tumor necrosis factor (TNF), phorbol 12-myristate 13-acetate (PMA), or control medium, and were evaluated for activation status, reactive oxygen species (ROS) production, phagocytic activity, granular enzyme and neutrophil extracellular traps (NETs) release. Serum levels of matrix metalloproteinase-9 (MMP-9), myeloperoxidase (MPO), TNF, interleukin 23 (IL-23), and interleukin 17A (IL-17A) were measured by ELISA. Serum Citrullinated histone H3 (CitH3) was measured as a NET biomarker. Here, the authors discuss their interesting results, which show an exhausted *in vitro* phenotype of PSA PMNs, as compared to PMNs of HCs, despite their more activated state *in vivo*, highlighting their plasticity and multifaceted roles in PsA pathophysiology (7, 8).

The interpretation of functional plasticity challenges the design of drugs intended to improve the pathogenetic profile of diseases. Understanding the interactions between the innate and adaptive immune systems is essential for interpreting the immunophenotypic plasticity observed in both health and disease. May these and further studies help bridge gaps in understanding the spectrum of autoimmunity and autoinflammation through focused studies on cellular and molecular mechanisms.
